# Knowledge, Perception, and Awareness of the Specialty of Oral and Maxillofacial Surgery Among the Public and Medical Field in Hail, Saudi Arabia: A Cross-Sectional Study

**DOI:** 10.7759/cureus.64334

**Published:** 2024-07-11

**Authors:** Ibtihag S Elnaem, Albandri M Alghris, Layla H Alenzi

**Affiliations:** 1 Oral and Maxilofacial Surgery, University of Hail College of Dentistry, Hail, SAU; 2 Dental Surgery, University of Hail College of Dentistry, Hail, SAU

**Keywords:** omf, oral and maxillofacial surgery, general public, dental and medical professionals, awareness, hail, saudi arabia

## Abstract

Aim and background

Oral and maxillofacial surgery is a crucial medical specialty focused on diagnosing, treating, and preventing diseases and injuries affecting the oral cavity, jaw, and face. There is a lack of awareness about this field in the specific study area of hail. This study aimed to assess the knowledge, awareness, and perception of maxillofacial surgery among healthcare professionals and the public.

Methods

The study included 225 participants, consisting of dental and medical professionals and the general public in Hail, Saudi Arabia. Convenience sampling was employed, and participants needed to be at least 20 years old and residents of Hail. A validated online questionnaire, translated into Arabic, was used for data collection.

Results

A proportion (78.2%) of dental professionals were aware of the specialty of oral and maxillofacial surgery. Among the general population and most medical professionals, the most commonly associated procedures with maxillofacial surgery were the treatment of injuries, bone fractures, and morphological changes in the mouth, jaw, and face, reported by 85.8% of the participants. Furthermore, 60.9% of the participants believed that maxillofacial surgery was the appropriate referral for cleft lip correction.

Conclusion

The study highlighted significant knowledge about the role of oral and maxillofacial surgeons among the target population. Out of the 225 participants, 137 participants (60.9%) chose the specialty of maxillofacial surgery for surgical intervention to treat cleft lips. Moreover, 75.1% (n = 169) of the participants had previously heard about oral and maxillofacial surgery, while 24.9% (n = 56) had never heard of this specialty. The findings also showed that 189 participants were not aware that a maxillofacial surgeon is responsible for treating severe deformities that cannot be eliminated using orthodontics, such as growth disorders, hypoplasia and hypoplasia of the lower jaw, and asymmetry of the lower jaw (prognathism and retrognathism of the jaw). To bridge this knowledge gap, it is crucial to implement targeted educational initiatives and awareness campaigns among both the general population and healthcare professionals. By increasing awareness and understanding of the specialized role of oral and maxillofacial surgeons, patient care can be optimized, and appropriate referrals to these specialists can be ensured.

Clinical significance

This study shows the importance of increasing awareness of oral and maxillofacial surgery among health professionals and the general public. Improved understanding of this specialty can lead to better patient outcomes and early referrals.

## Introduction

Oral and maxillofacial surgery is one of the specialties of dentistry that includes diagnosis, treatment, prevention, and convalescence from diseases and injuries of the teeth, oral cavity, jaw, and face. It is not only limited to cosmetic purposes; it has developed into a recognized surgical specialist. It helps treat a variety of disorders pertaining to the mouth, jaw, and face. The specialization covers a wide range of conditions, including orofacial discomfort, orofacial infections, temporomandibular disorders, cancer, aesthetic surgery, cleft lip and palate, and salivary gland diseases [1]. Although the field of oral and maxillofacial surgery has growing quickly in Saudi Arabia, there does not seem to be enough precise knowledge about it[1,2]. While oral and maxillofacial surgery is a well-recognized specialty in many parts of the world, there is limited research on the understanding and awareness of this field among healthcare professionals in Hail, Saudi Arabia. This is an important knowledge gap, as the prevalence of oral and maxillofacial conditions in the region and their impact on patient outcomes highlight the need for improved awareness and understanding among dental and medical professionals. A comprehensive literature review reveals that although hospitals worldwide acknowledge oral and maxillofacial surgery as a well-recognized specialty of the facial skeleton, there are still differences in students', healthcare professionals', and laypersons' understanding of the field's extent and familiarity with oral and maxillofacial surgery in surgical procedures. However, it is likely not always realized how broad the specialty of oral and maxillofacial surgery is to the general public and many other healthcare providers [3-5]. The aim of this study is to assess the level of knowledge and understanding of oral and maxillofacial surgery among dental and medical professionals in Hail, Saudi Arabia.

## Materials and methods

Study design

A cross-sectional study was conducted through online and social media channels among the Saudi population between June 1, 2023, and August 23, 2023.

Study criteria (inclusion and exclusion)

This study aimed to involve dental and medical professionals and the general population in the city of Hail, Saudi Arabia. Participants must be at least 20 years old, male or female, and a resident of the Hail region in order to meet inclusion requirements. Those who are 19 years old or younger or do not reside in Hail are excluded from the study.

Data collection

Using an online sample size calculator [6], 225 individuals were selected with a confidence level of 95% and a confidence interval (CI) of 5%, and a non-response rate of 50% was factored into the sample size calculation to account for potential challenges in the participant recruitment and response, which is commonly observed in survey-based studies conducted in healthcare settings, and a population size of approximately 731,141 in Hail [7]. Convenience sampling was the method used, which depends on the availability of individuals and their willingness to participate. The study questionnaire was developed by the authors based on a thorough review of the relevant literature and input from expert clinicians in the field of oral and maxillofacial surgery (see Appendix). The questionnaire was assessed for content validity by a panel of subject matter experts and pilot-tested with a small sample of the target population to ensure clarity and comprehension of the questions. The reliability of the questionnaire was evaluated through test-retest analysis, which demonstrated acceptable levels of internal consistency and stability over time. The electronic self-response questionnaire that was used to collect data was verified. The questionnaire was then translated from English to Arabic using a standard forward-backward translation process. The translated version was reviewed by a panel of bilingual experts to ensure conceptual equivalence with the original English version.

Further pilot testing was conducted with a small sample of the target population to assess the clarity and comprehension of the Arabic questionnaire, and any necessary revisions were made prior to the final deployment of the survey. The survey was published through social media such as X, WhatsApp, Snapchat, and Instagram to obtain information from various regions within Hail. The questionnaire was organized into sections covering demographics and other clinical conditions that are special to the practice of oral and maxillofacial surgery, as shown in Table 1. The demographic component includes age and gender and asks if they are a health practitioner and if they have ever heard about the specialty of oral and maxillofacial surgery. The other section includes clinical conditions, and the participants were asked to select who would be most appropriate or competent in treating each clinical condition, with only one option available for each disease.

**Table 1:** Demographic distribution of the study population

Variables		n (%)
Gender	Male	30.7%
	Female	69.3%
Age groups	20-25	53.8%
	26-30	11.6%
	31-40	9.3%
	41-50	8.4%
	51-60	14.2%
	65 and above	2.7%

Data analysis

The data were entered and analyzed using IBM SPSS Statistics for Windows, version 28.0 (IBM Corp., Armonk, NY, USA). A descriptive analysis of the data was conducted, followed by inferential statistical tests. Categorical variables were compared using chi-square and Fisher's exact tests. Statistical significance was defined as a p-value ≤ 0.05 at the 95% CI.

The questions were arranged and analyzed according to their specialty (trauma, pathology, reconstruction, or cosmetic)[4]. The data were gathered using Google Sheets and Google Forms (Google LLC, Mountain View, California, USA). Microsoft Excel (Microsoft Corporation, USA) was used for data coding and processing, after which the data were imported into SPSS Statistics version 28 for analysis.

Ethics approval

This study has been reviewed and approved by the Research Ethics Committee (REC) at the University of Hail dated 2/10/2023 (research no. H-2023-362).

## Results

In this study, out of 225 participants, 169 (75.1%) had heard of the specialty of maxillofacial surgery, while 56 (24.9%) had not heard of it. Sixty-nine of them (30.7%) confirmed that the ear, nose, and throat specialty is concerned with the treatment of cleft lip, while 12 participants (5.2%) indicated that pediatrics is responsible for therapeutic intervention for cleft lip, and seven participants (3.1%) chose the Obstetrics and Gynecology Department as responsible for the treatment. While 137 participants (60.9%) chose the specialty of maxillofacial surgery for surgical intervention to treat the cleft lip, 193 participants (85.8%) believe that the specialty of maxillofacial surgery is concerned with treating bone injuries and fractures, deformities, and morphological changes of the mouth, jaw, and face. Thirteen participants (5.8%) indicated that the specialty of maxillofacial surgery is interested in knee joint replacement treatment. Eleven participants (4.9%) chose treating middle ear infections as the main focus of the maxillofacial surgery specialty, and eight participants (3.6%) indicated that the maxillofacial surgery specialty focuses on treating vocal cord paralysis.

Out of the 225 participants, 35 (15.6%) chose dermatology as their specialty for treating obstructive sleep apnea, 17 (7.6%) chose pediatrics for treating obstructive sleep apnea, and 18 (8%) chose pathology for obstetrics and gynecology, while 155 participants (68.9%) chose the specialty of maxillofacial surgery as the department specialized in treating obstructive sleep apnea. A total of 176 participants (78.2%) believed that the maxillofacial specialty was derived from dentistry, while 49 participants (21.8%) were not aware, as shown in Table 2 and ​​​​​​Table 3.

**Table 2:** Comparison of correct response knowledge scores based on the age group

Q. no.	Questions		Gender N (%)		Chi-square (2-sided)	
			Male	Female	Value	Asymp. Sig
Q1	Have you ever heard about the specialty of oral and maxillofacial surgery?	Disagree	60 (38.5%)	19 (27.5%)	6.849%	0.009%
		Agree	96 (61.5%)	50 (72.5%)		
Q2	Harelip is one of the common cases in the medical field; in your opinion, which department could treat it?	Department of Ear, Nose, and Throat	52 (33.3%)	17 (24.6%)	2.487%	0.478%
		Department of Pediatrics	7 (4.5%)	5 (7.2%)		
		Department of Obstetrics and Gynecology	4 (2.6%)	3 (4.3%)		
		Department of Oral and Maxillofacial Surgery	93 (59.6%)	44 (63.8%)		
Q3	Choose one of the cases below that you believe a maxillofacial surgeon could treat.	Injuries, bone fractures, deformities, and morphological changes of the mouth, jaw, and face	133 (85.3%)	60 (87.0%)	2.910%	0.406%
		Knee joint replacement	11 (7.1%)	2 (2.9%)		
		Treatment of middle ear infection	8 (5.1%)	3 (4.3%)		
		Treatment of vocal cord paralysis	4 (2.6%)	4 (5.8%)		
Q4	You might have heard about obstructive sleep apnea; which department do you think could treat such cases?	Dermatologist	30 (19.2%)	5 (7.2%)	7.603%	0.055%
		Department of Pediatrics	13 (8.3%)	4 (5.8%)		
		Department of Obstetrics and Gynecology	14 (9.0%)	4 (5.8%)		
		Department of Oral and Maxillofacial Surgery	99 (63.5%)	56 (81.2%)		
Q5	Do you know that oral and maxillofacial surgery is one of the dental specialties?	Yes	125 (80.1%)	51 (73.9%)	2.800%	0.731%
		No	31 (19.9%)	18 (26.1%)		
Q6	Do you think that a maxillofacial surgeon is concerned with the diagnosis and treatment of cysts and tumors in the head and neck?	Yes	88 (56.4%)	48 (69.6%)	3.463%	0.063%
		No	68 (43.6%)	21 (30.4%)		
Q7	Who is the doctor responsible for treating severe deformities that cannot be eliminated by using orthodontics, such as growth disorders, hypoplasia, hypoplasia, and mandibular asymmetry "prognathism or retrognathism"?	Orthodontist	48 (30.8%)	16 (23.2%)	9.369%	0.009%
		Internal medicine	22 (14.1%)	2 (2.9%)		
		Maxillofacial surgeon	86 (55.1%)	51 (73.9%)		
Q8	Do you think that dealing with craniosynostosis is a part of the nature of the specialty of maxillofacial surgery?	Yes	62 (39.7%)	26 (37.7%)	0.209%	0.901%
		No	29 (18.6%)	12 (17.4%)		
		I don’t know	65 (41.7%)	31 (44.9%)		
Q9	Studies have shown that the most common tumor in children is melioblastoma. In your opinion, what is the most appropriate doctor to treat this condition?	Dermatologist	51 (32.9%)	5 (7.5%)	17.655%	0.001%
		ENT doctor	25 (16.1%)	11 (16.4%)		
		Maxillofacial surgeon	74 (47.7%)	46 (68.7%)		
		Orthodontist	5 (3.2%)	5 (7.5%)		
Q10	Orthognathic surgery, commonly known as jaw surgery, is a procedure that straightens out your lower (mandible) and upper (maxilla) jaws. Your bite may suffer if your jaws are not in alignment, which will also make it challenging for you to speak and eat. Who do you believe could perform the procedure?	Department of OMFS	115 (74.2%)	57 (82.6)	1.931%	0.381%
		Orthodontist	18 (11.6%)	5 (7.2%)		
		Internal Medicine	22 (14.2%)	7 (10.1%)		
Q11	One of the cosmetic operations is lip, cheek, and chin augmentation. Which department do you think could treat such cases?	Department of OMFS	60 (38.5%)	35 (50.7%)	3.024%	0.388%
		Department of Dermatology	23 (14.7%)	8 (11.6%)		
		Department of Plastic Surgery	66 (42.3%)	23 (33.3%)		
		Department of ENT	7 (4.5%)	3 (4.3%)		

**Table 3:** Comparison of correct response knowledge scores based on gender

Q. no.	Questions	Choices	Age group						Chi-square (2-sided)	
			20-25	26-30	31-40	41-50	51-60	65+	Value	Asymp. Sig
Q1	Have you ever heard about the specialty of oral and maxillofacial surgery?	Disagree	104 (86%)	15 (57.7%)	15 (71.4%)	11 (57.9%)	20 (62.5%)	4 (66.7%)	17.941%	.003%
		Agree	17 (14%)	11 (42.3%)	6 (28.6%)	8 (42.1%)	12 (37.5%)	2 (33.3%)		
Q2	Harelip is one of the common cases in the medical field; in your opinion, which department could treat it?	Department of Ear, Nose and Throat	33 (27.3%)	10 (38.5%)	10 (47.6%)	6 (31.6%)	9 (28.1%)	1 (16.7%)	26.121%	.037%
		Department of Pediatrics	4 (3.3%)	5 (19.2%)	0 (0.0%)	0 (0.0%)	3 (9.4%)	0 (0.0%)		
		Department of Obstetrics and Gynecology	3 (2.5%)	1 (3.8%)	1 (4.8%)	2 (10.5%)	0 (0.0%)	0 (0.0%)		
		Department of Oral and Maxillofacial Surgery	81 (66.9%)	10 (38.5%)	10 (47.6%)	11 (57.9%)	20 (62.5%)	5 (83.3%)		
Q3	Choose one of the cases below that you believe that a maxillofacial surgeon could treat.	Injuries, bone fractures, deformities, and morphological changes of the mouth, jaw, and face	105 (86.8%)	19 (73.1%)	18 (85.7%)	18 (94.7%)	30 (93.8%)	3 (50.0%)	16.967%	.321%
		Knee joint replacement	6 (5.0%)	3 (11.5%)	2 (9.5%)	1 (5.3%)	0 (0.0%)	1 (16.7%)		
		Treatment of middle ear infection	6 (5.0%)	3 (11.5%)	0 (0.0%)	0 (0.0%)	1 (3.1%)	1 (16.7%)		
		Treatment of vocal cord paralysis	4 (3.3%)	1 (3.8%)	1 (4.8%)	0 (0.0%)	1 (3.1%)	1 (16.7%)		
Q4	You might have heard about obstructive sleep apnea; which department do you think could treat such cases?	Dermatologist	22 (18.2%)	6 (23.1%)	4 (19.0%)	0 (0.0%)	3 (9.4%)	0 (0.0%)	19.975%	.173%
		Department of Pediatrics	9 (7.4%)	2 (7.7%)	2 (9.5%)	2 (10.5%)	2 (6.3%)	0 (0.0%)		
		Department of Obstetrics and Gynecology	6 (5.0%)	5 (19.2%)	3 (14.3%)	3 (15.8%)	1 (3.1%)	0 (0.0%)		
		Department of Oral and Maxillofacial Surgery	84 (69.4%)	13 (50.0%)	12 (57.1%)	14 (73.7%	26 (81.3%)	6 (100%)		
Q5	Do you know that oral and maxillofacial surgery is one of the dental specialties?	Yes	96 (79.3%)	21 (80.8%)	18 (85.7%)	13 (68.4%)	23 (71.9%)	5 (83.3%)	2.800%	.731%
		No	25 (20.7%)	5 (19.2%)	3 (14.3%)	6 (31.6%)	9 (28.1%)	1 (16.7%)		
Q6	Do you think that a maxillofacial surgeon is concerned with the diagnosis and treatment of cysts and tumors in the head and neck?	Yes	62 (51.2%)	23 (88.5%)	16 (76.2%)	12 (63.2%)	20 (62.5)	3 (50.0%)	15.390%	.009%
		No	59 (48.8%)	3 (11.5%)	5 (23.8%)	7 (36.8%)	12 (37.5%)	3 (50.0%)		
Q7	Who is the doctor responsible for treating severe deformities that cannot be eliminated by using orthodontics, such as growth disorders, hypoplasia and hypoplasia, and mandibular asymmetry "prognathism or retrognathism"?	Orthodontist	35 (28.9%)	8 (30.8%)	6 (28.6%)	6 (31.6%)	8 (25.0%)	1 (16.7%)	13.004%	.223%
		Internal medicine	13 (10.7%)	6 (23.1%)	4 (19.0%)	1 (5.3%)	0 (0.0%)	0 (0.0%)		
		Maxillofacial surgeon	73 (60.3%)	12 (46.2%)	11 (52.4%)	12 (63.2%)	24 (75.0%)	5 (83.3%)		
Q8	Do you think that dealing with craniosynostosis is part of the nature of the specialty of maxillofacial surgery?	Yes	50 (41.3%)	11 (42.3%)	8 (38.1%)	5 (26.3%)	11 (34.4%)	3 (50.0%)	16.264%	.092%
		No	15 (12.4%)	9 (34.6%)	7 (33.3%)	5 (26.3%)	5 (15.6%)	0 (0.0%)		
		I don’t know	56 (46.3%)	6 (23.1%)	6 (28.6%)	9 (47.4%)	16 (50.0%)	3 (50.0%)		
Q9	Studies have shown that the most common tumor in children is melioblastoma. In your opinion, what is the most appropriate doctor to treat this condition?	Dermatologist	37 (30.8%)	8 (32.0%)	6 (28.6%)	2 (10.5%)	2 (6.3%)	1 (20.0%)	21.976%	.108%
		ENT doctor	16 (13.3%)	7 (28.0%)	5 (23.8%)	3 (15.8%)	5 (15.6%)	0 (0.0%)		
		Maxillofacial surgeon	63 (52.5%)	10 (40.0%)	9 (42.9%)	13 (68.4%)	22 (68.8%)	3 (60.0%)		
		Orthodontist	4 (3.3%)	0 (0.0%)	1 (4.8%)	1 (5.3%)	3 (9.4%)	1 (20.0%)		
Q10	Orthognathic surgery, commonly known as jaw surgery, is a procedure that straightens out your lower (mandible) and upper (maxilla) jaws. Your bite may suffer if your jaws are not in alignment, which will also make it challenging for you to speak and eat. Who do you believe could perform the procedure?	Department of OMFS	95 (78.5%)	16 (61.5%)	15 (75.0%)	14 (73.7%)	27 (84.4%)	5 (83.3%)	17.850%	.058%
		Orthodontist	14 (11.6%)	1 (3.8%)	1 (5.0%)	3 (15.8%)	4 (12.5%)	0 (0.0%)		
		Internal medicine	12 (9.9%)	9 (3.8%)	4 (20.0%)	2 (10.5%)	1 (3.1%)	1 (16.7%)		
Q11	One of the cosmetic operations is lip, cheek, and chin augmentation. Which department do you think could treat such cases?	Department of OMFS	43 (35.5%)	13 (50.0%)	10 (47.6%)	9 (47.4%)	17 (53.1%)	3 (50.0%)	22.151%	.104%
		Department of Dermatology	16 (13.2%)	5 (19.2%)	2 (9.5%)	2 (10.5%)	6 (18.8%)	0 (0.0%)		
		Department of Plastic Surgery	59 (48.8%)	4 (15.4%)	8 (38.1%)	7 (36.8%)	8 (25.0%)	3 (50.0%)		
		Department of ENT	3 (2.5%)	4 (15.4%)	1 (4.8%)	1 (5.3%)	1 (3.1%)	0 (0.0%)		

Based on the gender of the participants, the chi-square value for people who have heard of the specialty of maxillofacial surgery is 0.009%, the chi-square value for those who thought that maxillofacial surgery falls under the dental specialty is 0.731%, while the chi-square value for those who thought that maxillofacial surgery is concerned with diagnosing and treating cyst is 0.063%. The values ​​based on age were 0.003% for those who had heard of maxillofacial surgery. A question arose: “Do you know that oral and maxillofacial surgery is one of the dental specialties?” Those who believed that the maxillofacial surgeon is concerned with treating the cyst based on age were 0.09%.

Out of 225 participants, 136 (60.4%) believed that maxillofacial surgeons care about diagnosing and treating cysts and tumors in the head and neck, while 89 of them (39.6%) were not in agreement. Sixty-four participants (28.4%) believed that the orthodontist is the doctor responsible for treating severe deformities that cannot be eliminated with orthodontics, such as growth disorders, hypoplasia and hypoplasia of the lower jaw, and asymmetry of the lower jaw, ”prognathism or retrognathism.” Twenty-four participants (10.7%) believed that the Department of Internal Medicine was responsible, and 137 participants (60.9%) chose the Department of Maxillofacial Surgery as the department responsible for these cases. Eighty-eight participants (39.1%) believed that dealing with craniosynostosis is part of the nature of the specialty of maxillofacial surgery, while 41 participants (18.2%) were not in agreement, while 96 participants (42.7%) were not aware of the answer. Of the 225 participants, 56 (25.2%) believed that the dermatologist was the person involved in the treatment of meloblastoma, while 36 (16.2%) chose the ENT doctor as the person involved in the treatment. One-hundred twenty participants (54.1%) chose the maxillofacial surgeon, while 10 participants (4.5%) chose the orthodontist. A total of 172 participants (75.8%) believed that orthognathic surgery, commonly known as jaw surgery, is a procedure performed in the Department of Maxillofacial Surgery, while 23 participants (10.3%) chose the orthodontist as responsible for these cases. Twenty-nine participants (12.9%) chose internal medicine as the responsible department. Ninety-five participants (42.2%) indicated that lip, cheek, and chin augmentation procedures are performed in the maxillofacial surgery department. Thirty-one participants (13.8%) chose the Dermatology Department as the department responsible for these procedures, while 89 participants (39.6%) chose the Department of Plastic Surgery for these procedures. Finally, 10 participants (4.4%) chose the Department of Ear, Nose, and Throat as the relevant department for these procedures.

The mean responses show that the participants had varying levels of knowledge about the specialty of maxillofacial surgery. On average, participants scored 2.26 out of 5 on questions related to age, 1.31 out of 5 on gender-related questions, and 1.25 out of 5 on whether they had heard of maxillofacial surgery. For questions assessing their understanding of specific maxillofacial surgery cases and treatments, the mean responses ranged from 1.22 out of 5 on whether they knew maxillofacial surgery is a dental specialty, up to 3.30 out of 5 on which department they thought could treat obstructive sleep apnea. The mean responses indicate there are significant gaps in participant knowledge about the scope and capabilities of the maxillofacial surgery specialty as shown in Table 4.

**Table 4:** Mean score of the responses

Questions	Mean
Age	2.26
Gender	1.31
Have you ever heard about the specialty of oral and maxillofacial surgery?	1.25
Harelip is one of the common cases in the medical field; in your opinion, which department could treat it?	2.94
Choose one of the cases below that you believe a maxillofacial surgeon could treat.	1.26
You might have heard about obstructive sleep apnea, but which department do you think could treat such cases?	3.30
Do you know that oral and maxillofacial surgery is one of the dental specialties?	1.22
Do you think that a maxillofacial surgeon is concerned with the diagnosis and treatment of cysts and tumors in the head and neck?	1.40
Who is the doctor responsible for treating severe deformities that cannot be eliminated by using orthodontics, such as growth disorders, hypoplasia and hypoplasia, and mandibular asymmetry "prognathism or retrognathism"?	2.32
Do you think that dealing with craniosynostosis is part of the nature of the specialty of maxillofacial surgery?	2.04
Studies have shown that the most common tumor in children is melioblastoma. In your opinion, what is the most appropriate doctor to treat this condition?	2.38
Orthognathic surgery, commonly known as jaw surgery, is a procedure that straightens out your lower (mandible) and upper (maxilla) jaws. Your bite may suffer if your jaws are not in alignment, which will also make it challenging for you to speak and eat.	1.49
One of the cosmetic operations is lip, cheek, and chin augmentation; which department do you think could treat such cases ?	2.06

## Discussion

The present study aimed to investigate the knowledge of oral and maxillofacial surgeon roles among the public and healthcare professionals. The findings revealed that 42.7% of the participants reported limited knowledge about the role of oral and maxillofacial surgeons, while 32.9% reported that they had no knowledge about the role of oral and maxillofacial surgeons. Moreover, 8.4% reported that they had excellent knowledge about oral and maxillofacial surgeon roles. The findings from this study offer meaningful information about the level of understanding and knowledge regarding oral and maxillofacial surgery among the intended audience.

The findings indicate that 42.7% of the participants reported limited knowledge about the role of oral and maxillofacial surgeons. This suggests a lack of familiarity with the specific responsibilities and expertise of these specialists. The limited knowledge might be due to various factors, such as insufficient exposure to oral and maxillofacial surgery during formal education or limited awareness campaigns targeting the general population. These results align with previous studies that, according to the previous studies, out of 100, only 41% of the medical students, 76% of the medical practitioners, and 58% of the public have heard the name of the specialty [1].

In this study, 42.7% of the participants reported limited knowledge about the role of oral and maxillofacial surgeons, 32.9% reported that they had no knowledge about the role of oral and maxillofacial surgeons, and 8.4% reported that they had excellent knowledge about oral and maxillofacial surgeon roles. Compared to the previous studies by Hunter et al. (1996), only 41% of medical students, 76% of medical practitioners, and 58% of the public had heard of the oral and maxillofacial surgery specialty [1]. Alnofaie et al. (2019) assessed knowledge, awareness, and perception of oral and maxillofacial surgery among the public and professionals in Saudi Arabia [2]. O'Connor et al. (2015) reviewed recent advances in the management of oral and maxillofacial trauma [3]. Kamal et al. (2021) examined knowledge, attitude, and perception of oral and maxillofacial surgery among healthcare professionals and the public in a Gulf Cooperation Council country[4]. Laskin et al. (2002) looked at public recognition of specialty designations, including oral and maxillofacial surgery[5]. Both this study and the previous research consistently found a lack of knowledge and awareness about the roles and responsibilities of oral and maxillofacial surgeons among both the public and healthcare professionals. The specific percentages from your study provide more detailed insights into the level of knowledge compared to the general findings reported in the earlier studies. Collectively, these studies highlight the need for better education and outreach efforts to improve understanding. The specialty of oral and maxillofacial surgery is of great importance.

Study limitations

One key strength of the study methodology was the use of both quantitative and qualitative data collection, including questionnaires and open-ended responses. This mixed-methods approach allowed for a richer understanding of the nuances in participants' knowledge, attitudes, and beliefs surrounding oral and maxillofacial surgery. In addition, the study utilized well-established statistical techniques, such as chi-square and Fisher's exact tests, to rigorously analyze the data and identify statistically significant findings. This analytical rigor adds to the credibility and reliability of the results.

However, the authors acknowledge certain limitations that should be noted when evaluating the results. First, healthcare professionals, such as physicians and dentists, had a smaller sample size than public participation. While including both the general population and healthcare professionals allowed for a more comprehensive examination of awareness and views of oral and maxillofacial surgery, the disproportionate representation of these two groups may limit the capacity to make direct comparisons. Future research should attempt to recruit a more balanced sample to ensure equitable representation of both populations. Furthermore, this study used a cross-sectional methodology, which provides a snapshot of current knowledge, perceptions, and awareness at a certain point in time. Longitudinal studies that track changes over time would be beneficial.

## Conclusions

The study highlighted significant knowledge about the role of oral and maxillofacial surgeons among the target population. Out of the 225 participants, 137 participants (60.9%) chose the specialty of maxillofacial surgery for surgical intervention to treat cleft lips. In addition, 75.1% (n = 169) of the participants had previously heard about oral and maxillofacial surgery, while 24.9% (n = 56) had never heard of this specialty. The findings also showed that 189 participants were not aware that a maxillofacial surgeon is responsible for treating severe deformities that cannot be eliminated using orthodontics, such as growth disorders, hypoplasia and hypoplasia of the lower jaw, and asymmetry of the lower jaw (prognathism and retrognathism of the jaw). To bridge this knowledge gap, it is crucial to implement targeted educational initiatives and awareness campaigns among both the general population and healthcare professionals. By increasing awareness and understanding of the specialized role of oral and maxillofacial surgeons, patient care can be optimized, and appropriate referrals to these specialists can be ensured.

## Appendices

Study questionnaire

1- Do you agree to participate in this study? 

A) (agree)

B) (disagree)

2- Are you a health practitioner?

A) (agree)

B) (disagree)

3-Age 

A) 20-24

B) 25-30

C) 31-45

D) 46-50

E) 51-60

F) 65 and above

4- Gender

A) Female 

B) Male

5- Have you ever heard about the specialty of oral and maxillofacial surgery?

A) Yes 

B) No 

6- Harelip is one of the common cases in the medical field; in your opinion, which department could treat it?

A) Department of Ear, Nose, and Throat

B) Department of Paediatrics

C) Department of Obstetrics and Gynaecology

D) Department of Oral and Maxillofacial Surgery

7- Choose one of the cases below that you believe a maxillofacial surgeon could treat.

A) Injuries, bone fractures, deformities, and morphological changes of the mouth, jaw, and face

B) Knee joint replacement

C) Treatment of middle ear infection

D) Treatment of vocal cord paralysis

8- You might have heard about obstructive sleep apnea; which department do you think could treat such cases?

A) Dermatologist

B) Department of Paediatrics

C) Department of Obstetrics and Gynaecology

D) Department of Oral and Maxillofacial Surgery

9- Do you know that oral and maxillofacial surgery is one of the dental specialties?

A) Yes

B) No

10- Do you think that a maxillofacial surgeon is concerned with the diagnosis and treatment of cysts and tumors in the head and neck?

A) Yes

B) No

11- Who is the doctor responsible for treating severe deformities that cannot be eliminated by using orthodontics, such as growth disorders, hypoplasia and hypoplasia, and mandibular asymmetry "prognathism or retrognathism"?

A)‏ Orthodontist

B) Internal medicine

C) Maxillofacial surgeon

12- Do you think that dealing with craniosynostosis is part of the nature of the specialty of maxillofacial surgery?

A) Yes

B) No

C) I don't know

13- Studies have shown that the most common tumor in children is medulloblastoma. In your opinion, what is the most appropriate doctor to treat this condition?

A) Dermatologist

B) ENT doctor

C) Maxillofacial surgeon

D) Orthodontist

14- Orthognathic surgery, commonly known as jaw surgery, is a procedure that straightens out your lower (mandible) and upper (maxilla) jaws. Your bite may suffer if your jaws are not in alignment, which will also make it challenging for you to speak and eat.  Who do you believe could perform the procedure?

A) Department of Oral and Maxillofacial Surgery

B) Orthodontist

C) Department of Ear, Nose and Throat

D) Internal medicine

15- One of the cosmetic operations is lip, cheek, and chin augmentation; which department do you think could treat such cases?

A) Department of Oral and Maxillofacial Surgery

B) Department of Dermatology

C) Department of Plastic Surgery

D) Department of Ear, Nose, and Throat

16- How would you rate your knowledge of oral and maxillofacial surgery?

A) No knowledge

B) Limited knowledge

C) Good knowledge

D) Excellent knowledge
